# Reference‐Guided Chromosome‐Scale Genome Assembly With Insights on Population Genomics of the Atlantic Goliath Grouper (*Epinephelus itajara*), Islas del Rosario, Colombia

**DOI:** 10.1002/ece3.74323

**Published:** 2026-09-10

**Authors:** Juan Esteban Jácome Castro, José Gregorio Martínez, Jaime Rojas, Nadine Hakim, Diego Alejandro Quiroga, Jorge Duitama, Susana Caballero

**Affiliations:** ^1^ Laboratorio de Ecología Molecular de Vertebrados Acuáticos, LEMVA, Departamento de Ciencias Biológicas Universidad de los Andes Bogotá Colombia; ^2^ Grupo de Investigación Biociencias, Facultad de Ciencias de la Salud Institución Universitaria Colegio Mayor de Antioquia Medellín Colombia; ^3^ Oceanario Islas del Rosario—Centro de Investigaciones, Educación y Recreación de las Islas del Rosario (CEINER) Cartagena Colombia; ^4^ Systems and Computing Engineering Department Universidad de los Andes Bogotá Colombia; ^5^ Aquatic Vertebrate Integrative Conservation Laboratory, Department of Marine and Environmental Sciences Halmos College of Arts and Sciences, Nova Southeastern University Guy Harvey Oceanographic Center Dania Beach Florida USA

**Keywords:** aquaculture, comparative genomics, conservation genomics, *Epinephelus itajara*, genomic structure, Islas del Rosario

## Abstract

*Epinephelus itajara* , commonly known as the Atlantic Goliath grouper, is the largest species among the western North Atlantic groupers and is critically endangered. This species plays a crucial ecological, cultural, and economic role and has been the focus of captive breeding efforts at the Oceanario of the Rosario Islands, Colombia. However, despite its ecological and conservation importance, genomic resources and population genomic data for *E. itajara* remain scarce, particularly in the Colombian Caribbean. This study presents a reference‐guided chromosome‐scale genome assembly and an analysis of the population genomic structure of *E. itajara* using PacBio HiFi sequencing and Illumina technologies. The assembled genome has a total size of 1.12 Gb, with a contig N50 of 42.69 Mb and a scaffold N50 of 46.30 Mb. A total of 22,692 protein‐coding genes were identified after masking 46% of the genome, which consists of repetitive elements. Comparative genomic analyses revealed a high degree of collinearity with closely related *Epinephelus* species and identified *E. lanceolatus* as the closest relative, supporting recent divergence and conserved genome architecture within the genus. Additionally, a population genomics analysis was conducted using 7706 high‐quality SNPs to assess the genomic structure of captive populations. The results revealed four distinct genomic lineages, with moderate genetic differentiation among the sampled individuals. In the Colombian Caribbean, two unique lineages were identified, associated with the localities of Bahía Cispatá and Bahía Barbacoas, suggesting possible geographic isolation. These genomic resources provide valuable tools and new opportunities to better understand the genomic diversity, evolutionary history, and reproductive mechanisms of *E. itajara* . Moreover, they serve as a foundation for conservation strategies, including selective breeding programs aimed at increasing genomic diversity in captive populations and guiding restoration efforts in its natural habitat.

## Graphical abstract

We present a high‐quality chromosome‐level genome assembly and population genomic analysis of the critically endangered Goliath grouper ( *Epinephelus itajara* ) using PacBio HiFi and Illumina sequencing technologies. The 1.12 Gb genome assembly showed high contiguity and identified 22,692 protein‐coding genes, while comparative analyses revealed conserved genome architecture with closely related Epinephelus species. Population genomic analyses identified four distinct genomic lineages, including two unique lineages in the Colombian Caribbean, providing key resources for understanding genomic diversity and supporting conservation and captive breeding strategies.

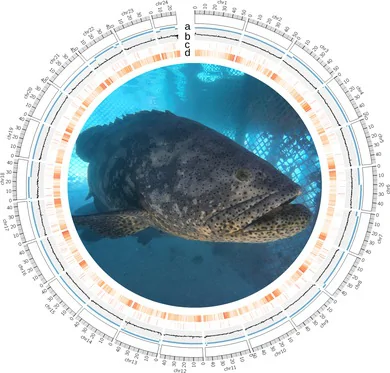

## 1 Introduction

The Atlantic goliath grouper ( *Epinephelus itajara* ) is the largest of the western North Atlantic groupers, reaching up to two meters in length and weighing approximately 400 kg (Bullock 1992; Freitas 2015; Frias‐Torres 2013). It belongs to the order *Perciformes* and exhibits slow growth, with males reaching sexual maturity between four and 6 years of age, while females mature between six and 7 years (Bullock 1992). During its juvenile stage, this species relies heavily on mangrove habitats as nursery areas before migrating to coral reefs, rocky formations, or even artificial structures such as sunken ships in adulthood (Silva‐Oliveira et al. 2008; Frias‐Torres 2013). A notable characteristic of the Goliath grouper is its strong site fidelity, as individuals tend to remain near their natal areas throughout their lifetimes (Freitas 2015; Mann et al. 2009; Murie et al. 2023; Pina‐Amargós and González‐Sansón 2009).

From an ecological perspective, the Goliath grouper occupies a high position in the trophic chain and it has been suggested as a top predator in coral reef ecosystems in the Caribbean Sea (Frias‐Torres 2013). While no natural predators are known for adult individuals, reports suggest that sharks occasionally prey on juveniles (Frias‐Torres 2006).

Beyond its ecological role, the Atlantic goliath grouper has historically supported recreational, subsistence, and small‐scale commercial fisheries throughout much of its distribution, representing an important resource for many coastal communities in the western Atlantic (Ferreira et al. 2014; Gerhardinger et al. 2006). Despite its ecological and economic importance, the Goliath grouper has been severely impacted by overfishing, leading to a drastic population decline. This is due to its slow growth, late sexual maturity, strong site fidelity, and vulnerability during spawning aggregations (Frias‐Torres 2013; Silva‐Oliveira et al. 2008). According to the International Union for Conservation of Nature (IUCN) Red List, the species is classified as vulnerable, with an estimated 33% decline in the total U.S. population between 1950 and 2014 (Bertoncini et al. 2018).

The evolutionary history of the *Epinephelus* genus reveals intriguing patterns of genetic differentiation and species diversification. *E. itajara* was once considered a single species distributed across the western Atlantic and eastern Pacific. However, mitochondrial and nuclear DNA analyses have identified important genetic differences between Atlantic and Pacific populations, with 3.5% divergence in the 16S gene (corresponding to an estimated separation of 2.9 million years) and 6.0% divergence in the cytochrome *b* gene (estimated at 3.0 million years). These findings support the classification of Goliath grouper into at least two distinct species: *E. itajara* in the Atlantic and *E. quinquefasciatus* in the Pacific. Additionally, within the western Atlantic, populations of *E. itajara* exhibit a pronounced pattern of genetic structuring, forming discrete population groups (Craig et al. 2009).

Recent assessments of animal genome sequencing have highlighted substantial geographic biases in the availability of genomic resources, with most reference genomes generated by institutions in the Global North (Álvarez et al. 2021). Expanding genomic resources to underrepresented regions is essential to obtain a more representative understanding of global biodiversity. For *E. itajara* , this gap is particularly evident in the Colombian Caribbean, where no genome assembly has previously been generated and the genetic composition of local populations has remained largely unexplored. To date, only 13 species have complete genome assemblies among the more than 150 grouper species distributed across 16 genera (Craig et al. 2011; Table S2). Previous studies have focused primarily on populations from Brazil and the United States, leaving an important geographic gap in the central portion of the geographical distribution of the species throughout the western Atlantic. A high‐quality genome for *E. itajara* would provide the foundation for genome‐wide conservation and breeding applications. First, it enables the discovery and precise localization of SNP markers throughout the genome, facilitating the identification of genomic regions associated with traits such as growth performance and immunity (Zhou et al. 2019). These genome‐wide markers can subsequently be used to estimate genomic relationships among broodstock, allowing the design of breeding strategies that maximize genetic diversity (Jerry et al. 2019). In addition, a genome assembly provides relevant information for the identification of loci associated with sex determination and other traits relevant to conservation programs and aquaculture efforts (Jiang et al. 2025).

Previous studies on the population genetic structure and variability of the goliath grouper ( *E. itajara* ) have primarily relied on mitochondrial DNA markers and microsatellites to assess population dynamics and genetic diversity. Some studies report significant genetic structuring among populations in Brazil (Silva‐Oliveira et al. 2008; Damasceno et al. 2015), often accompanied by low nucleotide diversity. Although this pattern may be consistent with the long‐term effects of overfishing and mangrove habitat loss, the relatively long generation time of the species suggests that current levels of genetic diversity may also reflect historical demographic processes. Additional work has confirmed this pattern of reduced genetic diversity despite relatively high levels of polymorphism (Benevides et al. 2014). Populations from northeastern Brazil, particularly Piauí and Rio Grande do Norte, exhibit some of the lowest levels of polymorphism (Benevides et al. 2014). Although classic markers are useful to obtain coarse information on genetic diversity, these markers have different limitations. Mitochondrial DNA represents only the maternal lineage, while microsatellites suffer from null alleles and variation in mutation patterns. In addition, both markers evolve rapidly, having a high mutation rate (Morin et al. 2004), and a high genotyping error rate (Al‐Samarai and Al‐Kazaz 2015). Consequently, these marker systems cannot fully characterize genome‐wide patterns of genetic diversity (Al‐Samarai and Al‐Kazaz 2015), provide high‐resolution population genomic analyses (Álvarez et al. 2021) or accurately estimate genomic relatedness (Al‐Samarai and Al‐Kazaz 2015). Although individual microsatellite loci are highly informative, thousands of genome‐wide SNPs provide substantially greater resolution for population genomic analyses and enable inferences across the entire genome, as has been shown in other studies for vertebrate species (Gallego‐García et al. 2021).

Accordingly, this study presents a *de novo* genome assembly for *E. itajara* . The genome assembly and its gene annotations were compared to available genomes of other species of *Epinephelus* to understand which level of genomic variation is present within the genus and to predict times of species divergence. Moreover, this work includes a SNP‐based characterization of the genetic diversity within a captive population from the Colombian Caribbean, aiming to obtain information on the amount and distribution of genetic diversity within this population. Both efforts provide genomic resources to support future conservation, captive breeding, and genetic management programs.

## 2 Methods

### 2.1 Collection Permits

The sample collection was carried out under the permission granted by the Parques Nacionales Natural (PNN) Corales del Rosario y de San Bernardo under the resolution No. 244 from October 2, 2024 (PIR 007‐2024).

### 2.2 Sample Collection

Eighteen individuals were sampled from the collection at the Oceanario, located within the Parque Nacional Natural Corales del Rosario and San Bernardo (PNN Corales del Rosario y de San Bernardo) at Islas del Rosario, Colombian Caribbean. Tissue samples were taken from the caudal fin of each individual. For the specimen selected for whole‐genome sequencing, a larger tissue sample (3 cm × 3 cm) was collected, whereas for the remaining individuals, smaller samples (1 cm × 1 cm) were taken. All samples were subsequently preserved in tubes containing 96% ethanol. Of the 18 individuals, 13 originated from Bahía Cispatá, Córdoba; three from Bahía Barbacoas, Bolívar; one from Ararca, Bolívar; and one from the surviving progeny of crosses conducted at the Oceanario (Table S1, ID: 60146) (Rojas et al. 2021).

### 2.3 DNA Extraction

DNA extraction was performed at the Laboratory of Molecular Ecology of Aquatic Vertebrates at Universidad de los Andes, Colombia. Two extraction kits were used: the Monarch HMW DNA Extraction Kit for Tissue (NEB #T3060S) was employed to extract high‐molecular‐weight genomic DNA from the individual selected for whole‐genome sequencing (ID 61517; Table S1). Additionally, DNA from this same individual was also extracted using the QIAGEN DNeasy Blood & Tissue Kit (Qiagen), which was used for the remaining samples (Table S1). DNA quality and quantity were assessed using Qubit and Nanodrop, and integrity was evaluated through electrophoresis on a 0.8% agarose gel.

### 2.4 Sequencing

One sample was sent to Macrogen (South Korea) for PacBio HiFi sequencing on the Revio system (Hon et al. 2020) and Illumina Whole Genome Sequencing (WGS) on the NovaSeq X platform (Modi et al. 2021) (ID 61517; Table S1). The remaining 17 samples were sent to SNPSaurus (United States) for Illumina WGS on the NovaSeq X platform (Table S1).

### 2.5 *Denovo* Assembly

First, the FASTQ files obtained from Macrogen (ID 61517; Table S1) underwent a quality assessment. Low‐quality reads and residual adapter sequences were removed using Fastp v0.23.2 (Chen 2023) with the following parameters for Illumina reads: “‐‐detect_adapter_for_pe ‐‐trim_poly_g ‐w 10 ‐‐trim_front1 13 ‐h.” Next, the filtered Illumina data was processed with Jellyfish v2.2.10 (Marcais and Kingsford 2011) to count *k*‐mers using the parameters “count ‐C ‐m 21 ‐s 23353736039.” The genome size was then estimated with Genomescope v2.0.1 (Vurture et al. 2017). For assembly, HifiAsm v0.16.1 (Cheng et al. 2021, 2022, 2024) was used with its default parameters. To minimize specific errors in the assembly, an error correction step was performed using the IndividualGenomeBuilder function of NGSEP v5.0.0 (Duitama et al. 2014; Gonzalez‐Garcia et al. 2023; Tello et al. 2019, 2023). For this, the raw PacBio reads were first mapped to the assembly with Minimap2 v2.24 (Li 2018) using the parameters “‐a ‐x map‐pb.” The resulting file was then sorted with Picard (Picard Toolkit 2019) using the following parameters: “SortSam MAX_RECORDS_IN_RAM = 1000000 SO = coordinate CREATE_INDEX = true I = /dev/stdin.” Finally, variant calling was performed with the SingleSampleVariantsDetector function of NGSEP v5.0.0 to generate a VCF file with the alignment file, which was subsequently used by the IndividualGenomeBuilder function to correct errors manifesting as homozygous variants. The resultant genome was polished following the pipeline suggested in the documentation of NextPolish2 v0.2.1 (Jiang et al. 2024). First, the Hifi reads were mapped against the genome using Minimap2 v2.24 (Li 2018) and then they were sorted and indexed using samtools v1.9 (Li et al. 2009) with the functions sort and index. Then, *K*‐mer datasets were produced from short‐read sequencing data with *k* sizes of 21, 31, 41, 51, and 61, through Yak (Li 2020). NextPolish2 was subsequently executed using the aligned reads as input.

To assemble the mitochondrial genome of the sequenced individual, the mitochondrial genome of *E. itajara* was downloaded from GenBank (access number: OP056827.1) (Padgett and Baeza 2025). A *blastn* alignment was then performed between the assembled genome and a database created with the *makeblastdb* function of BLAST v2.14 (Madden 2013) using the downloaded mitochondrial genome. This step allowed the removal of contigs that matched the reference. Next, another *blastn* alignment was carried out, this time using the raw PacBio reads against the previously sequenced mitochondrial genome, to perform a *de novo* assembly exclusively with those reads. For this, Unicycler v0.5.1 (Wick et al. 2017) was used with the parameter “‐‐start_genes,” specifying the first 500 bp of the downloaded genome to standardize the mitochondrial genome assembled in this study. The mitochondrial gene annotation was conducted with MitoZ v3.6 (Birney et al. 2004; Gertz et al. 2006; Juhling et al. 2012; Krzywinski et al. 2009; Li and Durbin 2009; Li et al. 2009; Meng et al. 2019; Nawrocki and Eddy 2013) using the parameter “annotate ‐‐clade Chordata”. This annotation was complemented by performing blastn searches of the features annotated in the sequence of OP056827.1 against the mitochondrial assembly. Lastly, the mitochondrial genome was incorporated into the assembly upon the matching contigs removal.

To generate a reference‐guided chromosome‐scale assembly, contigs corresponding to the mitochondrial genome were removed from the assembly. Scaffolding was performed based on collinearity with a closely related species. For this, RagTag v2.1.0 (Alonge 2022) was used with the parameter “scaffold ‐u.” The reference genome of *Epinephelus lanceolatus* (GCF_041903045.1) (Zhou et al. 2024), a species from the same genus, was employed to guide the scaffolding through synteny information. The Circos software (Krzywinski et al. 2009) was employed to illustrate the genomic characteristics of the assembly made in this study.

### 2.6 Technical Validation of the Genome Assembly

To assess the completeness of the genome, the Benchmarking Universal Single‐Copy Orthologues (BUSCO v5.3.2; Simão et al. 2015) software was used with the parameters “‐m genome ‐‐auto‐lineage‐euk ‐‐update‐data”, and QUAST v5.0.2 (Gurevich et al. 2013) was used with default parameters. Base‐level assembly accuracy and completeness were further evaluated using Merqury (Rhie et al. 2020). A meryl database was generated from the Illumina reads, and Merqury was run in non‐trio mode to estimate the consensus quality value (QV), *k*‐mer completeness, and *k*‐mer copy number spectra (Figure S4). To further assess assembly quality, PacBio HiFi reads were aligned back to the final assembly using Minimap2 v2.24 (Li 2018) with the map‐pb preset and the resulting alignments were sorted and indexed with Samtools v1.9 (Li et al. 2009). Read mapping statistics were obtained using samtools flagstat, and per‐base sequencing depth was computed using samtools depth to generate the coverage‐depth distribution (Figure S5). The *k*‐mer spectra, read‐depth distribution, and BUSCO duplication statistics were jointly inspected to identify evidence of uncollapsed haplotigs or redundant duplicated regions. Finally, to further evaluate the continuity of repetitive regions, the LTR Assembly Index (LAI) was calculated using the LAI script distributed with EDTA v2.2.1 (Ellinghaus et al. 2008; Gremme et al. 2013; Ou and Jiang 2019, 2018; Ou et al. 2019; Shi and Liang 2019; Su et al. 2019; Xiong et al. 2014; Xu and Wang 2007; Zhang et al. 2022). Intact LTR retrotransposons and whole‐genome repeat annotations generated during the EDTA pipeline were used as input to estimate assembly continuity within repetitive regions.

### 2.7 Genes and Transposable Elements Annotation

A *de novo* transposon (TE) annotation was performed using EDTA v2.2.1 (Ellinghaus et al. 2008; Gremme et al. 2013; Ou and Jiang 2019, 2018; Ou et al. 2019; Shi and Liang 2019; Su et al. 2019; Xiong et al. 2014; Xu and Wang 2007; Zhang et al. 2022) with the parameters “‐‐cds ‐‐sensitive 1 ‐‐anno 1 ‐‐evaluate 1”. The coding regions of *E. lanceolatus* (GCF_005281545.1) (Zhou et al. 2019) were used to remove gene fragments present within the transposons. The repetitive regions were masked using RepeatMasker v4.1.7‐p1 (Tarailo‐Graovac and Chen 2009) with the parameters “‐pa 8 ‐lib ‐gff ‐engine ncbi ‐html” and the TE library generated by EDTA v2.2.1. Gene annotation was carried out with Maker v3.01.04 (Cantarel et al. 2008) using the “‐fix_nucleotides” parameter, and the protein file of *E. lanceolatus* (GCF_005281545.1) (Zhou et al. 2019) was employed to identify homologous patterns. Finally, after removing the contigs corresponding to the mitochondrial genome from the original assembly, the assembled mitochondrial genome was incorporated.

### 2.8 Comparative Genomics

For this analysis, only chromosome‐level assemblies of the genus *Epinephelus* available in NCBI were selected. Assemblies that already included a valid GFF3 annotation file in the FTP repository were directly used, namely *E. moara* (GCF_006386435.1) (Zhou et al. 2021), *E. fuscoguttatus* (GCF_011397635.1) (Yang et al. 2022), and *E. akaara* (Dryad database: https://doi.org/10.5061/dryad.4398b9f) (Ge et al. 2019). Assemblies available in NCBI that lacked a valid annotation file were annotated de novo. These included *E. awoara* (GCA_035609425.1) (Zhang et al. 2024), *E. lanceolatus* (GCF_041903045.1) (Zhou et al. 2024), *E. polyphekadion* (GCA_041411175.1) (Li et al. 2024), *E. cyanopodus* (GCA_026686955.1) (Cao et al. 2022), and *E. tukula* (GCA_021130775.1) (Wang et al. 2022). Prior to annotation, each genome was masked using Repeat Masker v4.1.7‐p1 (Tarailo‐Graovac and Chen 2009) with the parameters “‐pa 8 ‐lib ‐gff ‐engine ncbi ‐html”, employing the TE library generated in this study. Gene annotation was then performed using MAKER v3.01.04 (Cantarel et al. 2008) with the parameter “‐fix_nucleotides”. For *E. awoara* , *E. polyphekadion* , and *E. tukula* , the protein dataset from *E. lanceolatus* (GCF_005281545.1) (Zhou et al. 2019) was used as homology evidence. The assembly of *E. lanceolatus* (GCF_041903045.1) (Zhou et al. 2024) was re‐annotated using its own protein dataset (GCF_005281545.1) (Zhou et al. 2019), because at the time of analysis no protein or GFF3 annotation was available for that assembly. In addition, this re‐annotation ensured methodological consistency, as this assembly was used as the reference during RagTag scaffolding due to its improved assembly statistics. Finally, the *E. cyanopodus* assembly (Cao et al. 2022) was annotated using the protein dataset from *E. akaara* (Ge et al. 2019), based on their phylogenetic proximity as reported by Cao et al. (2022).

The GenomesAligner function of NGSEP (Tello et al. 2023) was used to generate homologous gene clusters (orthogroups) and pairwise synteny blocks. Syntenic relationships were visualized using SynVisio (Bandi and Gutwin 2020). In addition, contig‐to‐assembly dot plots were generated in SynVisio to validate putative structural rearrangements identified in synteny analyses, particularly the inversion signal detected on chromosomes 4, 5, and 19. These dot plots were used to assess the consistency of the observed rearrangements across pairwise genome comparisons. For these comparisons, the original contigs contributing to the corresponding *E. itajara* chromosomes were used instead of the reference‐guided chromosomes. For visualization purposes, chromosomes from each species were manually reordered to match the chromosomal arrangement of *E. itajara* , which was used as the reference genome. This step facilitated direct comparison of collinearity patterns across species. In addition, chromosome orientations were manually adjusted when necessary to maximize syntenic alignment and improve interpretability of structural relationships. These adjustments reflect differences in chromosome assembly orientation and highlight the current lack of standardized chromosome naming and ordering across *Epinephelus* genomes. To improve clarity in multi‐species comparisons, synteny plots were generated by arranging *E. itajara* as a central reference genome, with one species displayed above and another below. A total of four comparative panels were produced to visualize all eight species.

The genomes aligner generates a presence/absence matrix with the counts of genes belonging to orthogroups within the genomes. These counts were used to identify the exact‐core genome as single‐copy genes present in all the genomes, also including genes with two or three copies in all the genomes. The remaining clusters were filtered to identify multicopy gene families as the families where at least one genome has more than two copies. To calculate nucleotide evolution statistics (Ks), the DNA coding sequences of single copy orthologs between each pair of genomes were aligned using a codon‐aware pairwise aligner available with the NGSEP distribution (class ngsep.transcriptome.CodonCDSPairwiseAlignment). Alignments were provided to the command codeml of paml v4.9j (Yang 2007) for estimation of ks proportions.

Approximate divergence times were inferred from Ks values based on divergence estimates previously reported for species within the genus *Epinephelus*. Considering published divergence ranges among closely related species in the genus (Yang et al. 2022; Craig et al. 2009; Zhou et al. 2021; Ma et al. 2016; Cao et al. 2022; Wang et al. 2022; Zhao et al. 2025), a proportional equivalence of approximately Ks = 0.01 to ~4 million years ago (Mya) was used as a tentative reference to contextualize relative divergence patterns among species. Divergence times for the remaining comparisons were extrapolated proportionally under the assumption of similar generation times among the analyzed species.

### 2.9 Population Structure Analysis

The reads from the whole genome sequenced with Illumina for a specimen of *E. itajara* (SRA: SRR21844067) from Port Canaveral, Florida, USA were downloaded. Prior to mapping, the Illumina reads were cleaned using Fastp v0.23.2 (Chen 2023) with the parameters “‐‐detect_adapter_for_pe ‐‐trim_poly_g ‐w 10 ‐‐trim_front1 19 ‐‐trim_tail1 3 ‐h”. The clean reads of 19 individuals were then aligned against the *E. itajara* assembly created in this study using Bowtie2 v2.4.5 (Langmead and Salzberg 2012; Langmead et al. 2009, 2019). First, an index was built using the bowtie2‐build function, which was then used to map the reads with Bowtie2 using the parameters “‐‐rg‐id ‐‐rg SM: ‐‐rg PL: ILLUMINA ‐x”. The resulting SAM file was then sorted using Picard (Picard Toolkit 2019) with the following parameters “SortSam MAX_RECORDS_IN_RAM = 000000 SO = coordinate CREATE_INDEX = true I = /dev/stdin”. Once the BAM file was obtained, it was used to call variants or SNPs via the MultisampleVariantsDetector function of NGSEP v5.0.0 (Duitama et al. 2014; Tello et al. 2019) with default parameters.

Multiple filters were applied to the resulting VCF to obtain high‐quality SNPs. The VCFFilter function of NGSEP v5.0.0 was used with the parameters “‐minMAF 0.05 ‐m 19 ‐q 40 ‐s”, where only SNPs genotyped in at least 19 individuals with a genotyping quality greater than 40, a minor allele frequency greater than 0.05, and biallelic SNPs were retained. Finally, to reduce linkage disequilibrium, a filter was applied using Vcftools v0.1.16 (The Variant Call Format and VCFtools et al. 2011), keeping only 1 SNP every 100 kb with the parameters “‐‐vcf ‐‐recode ‐‐recode‐INFO‐all ‐‐thin 100000”. SNPs passing these filters were used for population genomics analysis. First, to evaluate the population structure, a PCA was performed using the VCF2PCA software (Silva 2017). The VCF file was formatted to Structure format using PGDSpider v3.0.0.0 (Lischer and Excoffier 2012). The second approach was Bayesian and was implemented in STRUCTURE v2.3.4 (Evanno et al. 2005; Falush et al. 2003; Kopelman et al. 2015; Li and Liu 2018; Puechmaille 2016) under the admixture model with correlated allele frequencies. For each assumed number of clusters (*K* = 1–8), fifteen independent runs were performed with 500,000 Markov chain Monte Carlo (MCMC) iterations, discarding the first 50,000 as burn‐in. The optimal number of genetic clusters (*K*) was inferred using the estimators proposed by Puechmaille (2016), implemented in the StructureSelector web server (Li and Liu 2018). This approach was selected because it provides more reliable inference under uneven sampling conditions. The estimators evaluated included MedMedK, MaxMedK, MedMeanK, and MaxMeanK.

Finally, a distance matrix was calculated between the samples in the VCF prior to the VCFtools filter. A total of 32,813 SNPs were used in the VCFDistanceMatrixCalculator function of NGSEP to calculate the distance matrix, which was then used to generate a dendrogram with the HierarchicalClustering function of NGSEP and visualized in iTol (Letunic and Bork 2021).

### 2.10 Genetic Diversity Analyses

Estimating *H*_o_, *H*_e_, π, and pairwise *F*_ST_ requires that sampled individuals constitute a random‐mating population in Hardy–Weinberg equilibrium (HWE). When genetically distinct subpopulations are pooled, the Wahlund effect (1928) reduces the *H*_o_ from its expected value under HWE. Because population structure was detected in the dataset, the sampled individuals were not treated as a single panmictic unit. Accordingly, diversity metrics were estimated only within clusters with *n* ≥ 5 (Nei 1987; Nei and Roychoudhury 1974). Genetic groups represented by very few individuals were considered preliminary and were excluded from analyses requiring robust within‐group allele‐frequency estimation. All diversity index estimations, pairwise *F*_ST_, private allele counts, and kinship analyses were carried out via a custom python script using NumPy 2.2.6 (Harris et al. 2020) and Pandas 2.3.3 (McKinney 2010; The Pandas Development Team 2020). The script is available at the Supporting Information S1: *genetic_diversity_analysis.py*, and it takes as input the STRUCTURE‐format genotype matrix.

Observed heterozygosity (*H*_o_), expected heterozygosity (*H*_e_) (Nei 1973, 1987), and nucleotide diversity (π; Nei 1987; Tajima 1983) were computed per locus for the clusters with at least five individuals (*n* ≥ 5). Loci with any missing genotype in the focal cluster were excluded. Results were expressed as means ± SE across all polymorphic loci. Pairwise *F*_ST_ was estimated using the G_*ST*_ formulation of Nei (1987), which partitioned total expected heterozygosity (H*T*, from pooled allele frequencies) into within‐ (H*S*) and among‐cluster components; per‐locus *F*_ST_ values were averaged across all shared polymorphic loci, with SE derived from the locus‐level variance (Weir and Cockerham 1984). Pairwise comparisons were restricted to inferred genetic groups with sufficient sample size (*n* ≥ 5) because *F*_ST_ estimates become strongly biased when one or both groups contain very few individuals (Weir 1996; Meirmans and Hedrick 2011).

An allele was classified as private to a cluster if it occurred at non‐zero frequency exclusively in that cluster and was absent from all others. Loci with missing data in any cluster were excluded to prevent confounding absent genotyping with genuine allele absence. Private allele counts for groups represented by very few individuals were conservative underestimates, as detection was limited to alleles present in the sampled chromosomes.

Pairwise kinship coefficients (φ) among all 19 individuals were estimated using the genomic relationship matrix of VanRaden (2008). For each polymorphic locus with complete genotype data, allele dosage (0, 1, or 2 copies of the major allele) was centered against genome‐wide allele frequency, and the resulting variance–covariance matrix was scaled by the expected dosage variance; monomorphic loci were excluded. The φ coefficient measures the proportion of the genome shared identical‐by‐descent relative to the study population: values near zero indicate effectively unrelated pairs, values ≥ 0.125 are consistent with second‐degree relatives (e.g., half‐siblings), values approaching 0.25 indicate first‐degree kinship (full siblings or parent–offspring), and negative values reflect pairs sharing fewer alleles than the population‐wide baseline. Because this estimator operates at the individual level and requires neither Hardy–Weinberg equilibrium nor a minimum sample size, kinship was computed for all sampled individuals, including those belonging to genetic groups represented by a single individual, for which the diagonal element *G*ii provided the self‐kinship (inbreeding coefficient). Statistical computations were performed in Python 3.10.12 using NumPy 2.2.6 (Harris et al. 2020) and Pandas 2.3.3 (McKinney 2010; The Pandas Development Team 2020). Visualizations were produced with Matplotlib 3.10.9 (Hunter 2007) and Seaborn 0.13.2 (Waskom 2021) and included a color‐coded heatmap of the full pairwise kinship matrix with individuals ordered by cluster membership, and box‐and‐whisker plots with jittered individual data points showing the distribution of φ values within and between clusters.

## 3 Results

### 3.1 A New Genome Assembly for the Goliath Grouper

**FIGURE 1:**
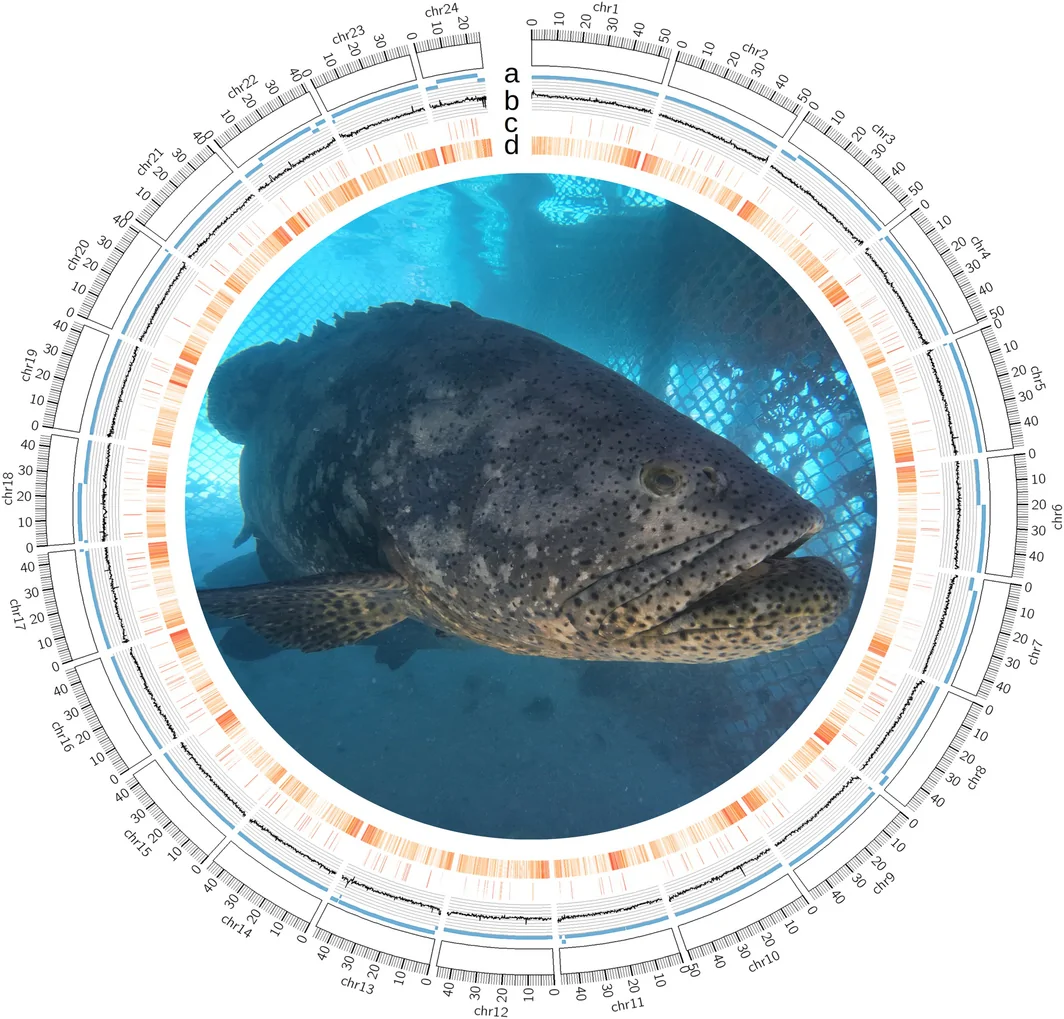
Genomic characteristics of *E. itajara*. (a) Alignment of contigs to the assembled pseudochromosomes. (b) GC content over 100 Kbp windows across the pseudochromosomes. (c) Density of single copy genes conserved in the *Actinopterygii* taxonomy. (d) Density of transposable elements. Photograph by Juan E Jácome.

The genome assembly was constructed using sequencing data from a single individual (ID 61517; Table S1) comprising 106 Gb of Illumina data and 91.4 Gb of PacBio data, with an average read depth of 94× and 81×, respectively. After quality filtering, all 91.4 Gb of PacBio Sequel II data were conserved, while 80 Gb of Illumina data was retained. Based on the 21‐mer analysis, the genome size was estimated to be around 1.01 Gbp with a 0.12% heterozygosity (Figure S1). The contig‐level assembly included 220 contigs with a median (N50) of 42.69 Mbp a maximum length of 54.24 Mbp and a total length of 1.12 Gbp. The reference‐guided scaffolding process reduced the total number of contigs to 168, with a median (N50) of 46.30 Mb, the longest contig account for 54.27 Mb and a total size of 1.12 Gbp. (Table 1). A subset of 76 contigs were merged into the 24 pseudochromosomes of the species. The remaining 143 unplaced contigs had a total length of ~20 Mb. Figure 1 displays the main features of the assembly. Chromosomes 1, 2, 10, 12, 14, 15, 16, 19, and 20 were built with only one contig, while the remaining chromosomes were composed of 2, 3, or 4 contigs (Figure 1a). The GC content in the genome is homogeneous across the chromosomes, with an average of 41.37% (Figure 1b). The assembly accuracy assessed with Merqury yielded a consensus quality value (QV) of 53.98 and a *k*‐mer completeness of 98.7%, indicating high consensus accuracy and recovery of the sequencing information (Table 1). Mapping of the PacBio HiFi reads back to the assembly resulted in a 100% primary alignment rate. The distribution of sequencing depth across the assembly showed a single dominant peak centered around the expected coverage, indicating a largely uniform read‐depth profile without major coverage anomalies (Figure S5). Likewise, the *k*‐mer copy number spectra generated by Merqury showed only a minimal proportion of duplicated *k*‐mers, providing no evidence of substantial uncollapsed haplotigs (Figure S4). The BUSCO analysis identified 3606 complete conserved genes (99.1%), including 3573 single‐copy (98.2%) and only 33 duplicated genes (0.9%), out of the 3640 Actinopterygii orthologs (Figure 1c and Figure S3). The assembly also achieved an LTR Assembly Index (LAI) of 3.18.

**TABLE 1:** Summary of main assembly statistics for the assembly at contig and scaffold levels.

Parameters	Contig	Scaffold
Number of contigs	220	168
Total size (Gb)	1.12	1.12
Largest contig (Mb)	54.24	54.27
GC (%)	41.37	41.37
N50 (Mb)	42.69	46.30
L50	12	12
Merqury (QV)	53.98	53.98
*k*‐mer completeness (%)	98.72	98.72
Read mapping rate (%)	100.00	100.00

The assembly includes a complete mitochondrial genome of *E. itajara*, with a total length of 16,752 bp. All features annotated in the publicly available mitochondrial genome of this species were identified in the new assembly. This includes a total of 13 protein‐coding genes, 2 ribosomal RNA genes (12S and 16S), and 22 transfer RNAs. The protein‐coding genes include subunits of NADH dehydrogenase (ND1, ND2, ND3, ND4, ND4L, ND5, ND6), cytochrome c oxidase (COX1, COX2, COX3), ATP synthase (ATP6, ATP8), and cytochrome b (CYTB). (Figure S2).

In total, 521 Mbp of repetitive sequences were masked, covering approximately 46.4% of the genome (Figure 1d). Retroelements accounted for 13.36% of the genome. Among these, LINEs make up 4.22%, and LTRs contribute 8.98%. Meanwhile, DNA transposons cover 28.44%, making them the most abundant class of transposable elements (TEs) in the analyzed genome. Overall, interspersed repeats comprise 44.53% of the genome. A higher density of TEs corresponds to a lower gene density, and vice versa.

### 3.2 Comparative Genomics

After transposable element masking, a total of 22,692 protein‐coding genes were predicted in the *E. itajara* genome. These genes were compared with annotated gene sets from eight species of the genus *Epinephelus* to assess synteny and structural conservation.

**FIGURE 2:**
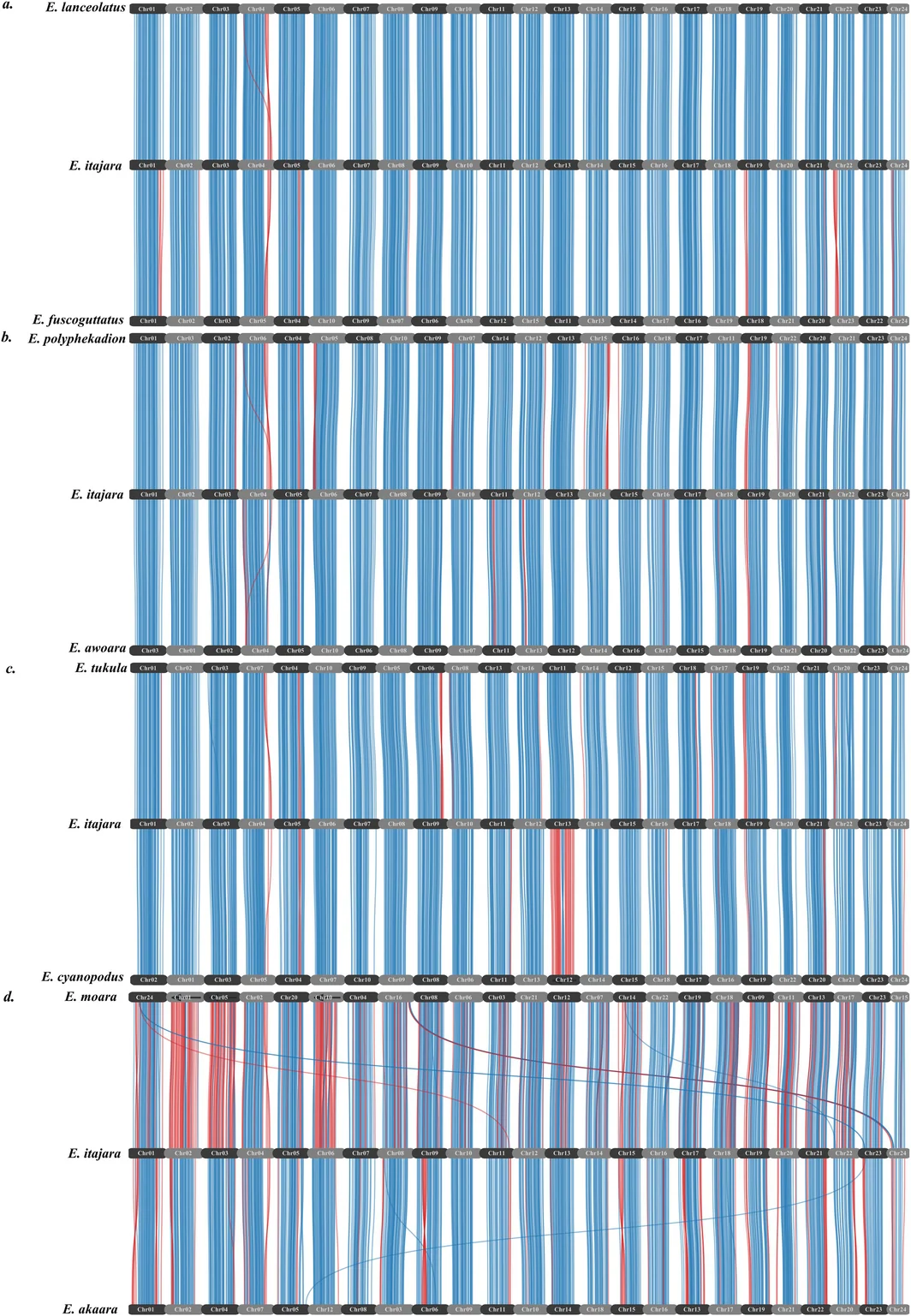
Synteny relationships between *E. itajara* and eight species of the genus *Epinephelus*. In each panel, *E. itajara* is shown as the central reference genome, with one species displayed above and another below for comparison. (a) Comparison of *E. lanceolatus* — *E. itajara* – *E. fuscoguttatus* . (b) Comparison of *E. polyphekadion* — *E. itajara* — *E. awoara* . (c) Comparison of *E. tukula* — *E. itajara* — *E. cyanopodus* . (d) Comparison of *E. moara*— *E. itajara* — *E. akaara* . Blue blocks indicate conserved synteny in the same orientation, while red blocks represent inverted regions. Chromosomes were reordered and reoriented relative to *E. itajara* to facilitate visualization of collinearity patterns.

Overall, chromosome number conservation and a high degree of collinearity were observed across most species when compared to *E. itajara* (Figure 2). Among them, *E. lanceolatus* exhibited the highest level of syntenic conservation (Figure 2a). We acknowledge that this structure conservation could be inflated because the *E. itajara* assembly was generated through reference‐guided scaffolding using *E. lanceolatus* . However, in this case, the bias produced by reference‐guided scaffolding is lessened by the fact that only 76 contigs were merged to assemble the 24 chromosomes (3.17 contigs per chromosome), and close to half of the chromosomes were assembled in a single contig (Figure 1A).

The most consistent putative structural signal involved chromosome 4 of *E. itajara* . A putative inversion was observed across all pairwise comparisons and was further supported by contig‐to‐assembly dot plots using the original contigs contributing to chromosome 4 of *E. itajara* (Figure S11). Additional recurrent putative inversion signals were detected on chromosomes 5 and 19 (Figures S12 and S13), which exhibited similar patterns across most of the analyzed *Epinephelus* species. For these chromosomes, contig‐to‐assembly dot plots were generated using the original contigs contributing to the corresponding *E. itajara* chromosomes. Less frequent putative inversion signals were identified on chromosomes 1, 9, and 11, being restricted to a subset of species (Figure 2).

Several chromosome reorientations were required to avoid false positive inversion calls due to lack of standards on chromosome orientation among the genomes. In *E. cyanopodus* , chromosome 12 was manually reoriented to match the *E. itajara* genome assembly (Figure 2c). Likewise, chromosomes 1, 5, and 10 of *E. moara* were manually reoriented. No chromosome reorientation was required for *E. akaara* (Figure 2d). After performing this curation process, *E. moara* and *E. akaara* exhibited the greatest degree of putative structural divergence relative to *E. itajara* . *E. moara* showed lower overall collinearity and numerous putative translocation events involving genes repositioned across different chromosomes. Similarly, *E. akaara* displayed multiple putative translocation signals together with several putative inversions distributed across the genome (Figure 2d).

**FIGURE 3:**
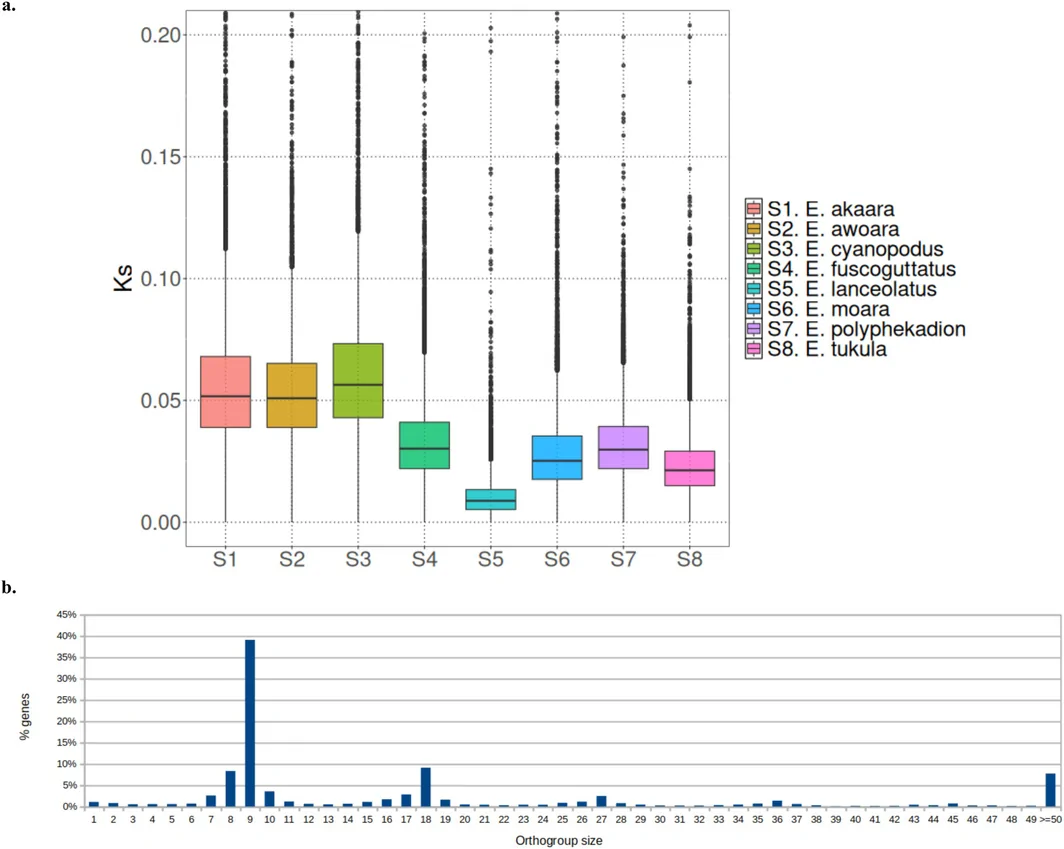
(a) Distribution of synonymous substitution rates (Ks) between *Epinephelus itajara* and eight species of the genus Epinephelus. (b) Distribution of orthogroup sizes across *Epinephelus itajara* and eight additional species of the genus *Epinephelus*.

A nucleotide evolution analysis was performed between pairs of single copy orthologs to estimate relative divergence times between *E. itajara* and the other species. The ratio of synonymous mutations (Ks) was on average 0.01 (Figure 3a) between *E. itajara* genes and *E. lanceolatus* genes suggesting that *E. lanceolatus* is the analyzed species closest to *E. itajara* . A group of four species (*E. fuscogutatus, E. moara, E. polyphekadion* and *E. tukula* ) had average *Ks* values between 0.02 and 0.03, (Figure 3a) suggesting that they form a clade from which *E. itajara* split about 10 million years ago (mya). Finally, another group of three species (*E. akaara, E. awoara* and *E. cyanopodus* ) had average Ks values above 0.05 (Figure 3a), suggesting that *E. itajara* split from these groups around 20 mya.

The annotated genes were also compared to the gene annotation for other eight species. A total of 205,348 gene annotations were clustered in 20,353 orthogroups (Figure 3b). From these, 8915 orthogroups (43.8%) covering 39.07% of the genes had a size corresponding to the number of analyzed species (nine). We verified that in 8685 of these groups, there was one gene copy for each species. The remaining groups of size 9 and the groups of lower size suggest the existence of gene presence/absence events between the species. Conversely, 4366 groups including 93,967 gene annotations had sizes larger than 9, evidencing different patterns of gene duplication. Peaks were observed in orthogroups of size 18, 27 and 36, which correspond to orthogroups formed by genes with two, three and four copies throughout the 9 genomes.

### 3.3 Genomic Diversity Within Captive Goliath Groupers

The sample used to assemble the genome was also processed with Illumina sequencing, generating approximately 80 Gbp of clean reads. Meanwhile, the raw reads from the 17 remaining samples accounted for about 28.6 Gb per sample, for an average read depth of 25.5×. Publicly available Illumina reads from an individual from Port Canaveral, Florida were also analyzed (SRA/GenBank accession: SRR21844067).

An average alignment percentage of 99.07% was achieved, confirming the high base pair quality of the assembly. Variant detection and genotyping from the aligned reads resulted in 3,096,820 SNPs that could be genotyped across the 19 individuals. A conservative filtering procedure resulted in 7706 SNPs used for population analysis.

**FIGURE 4:**
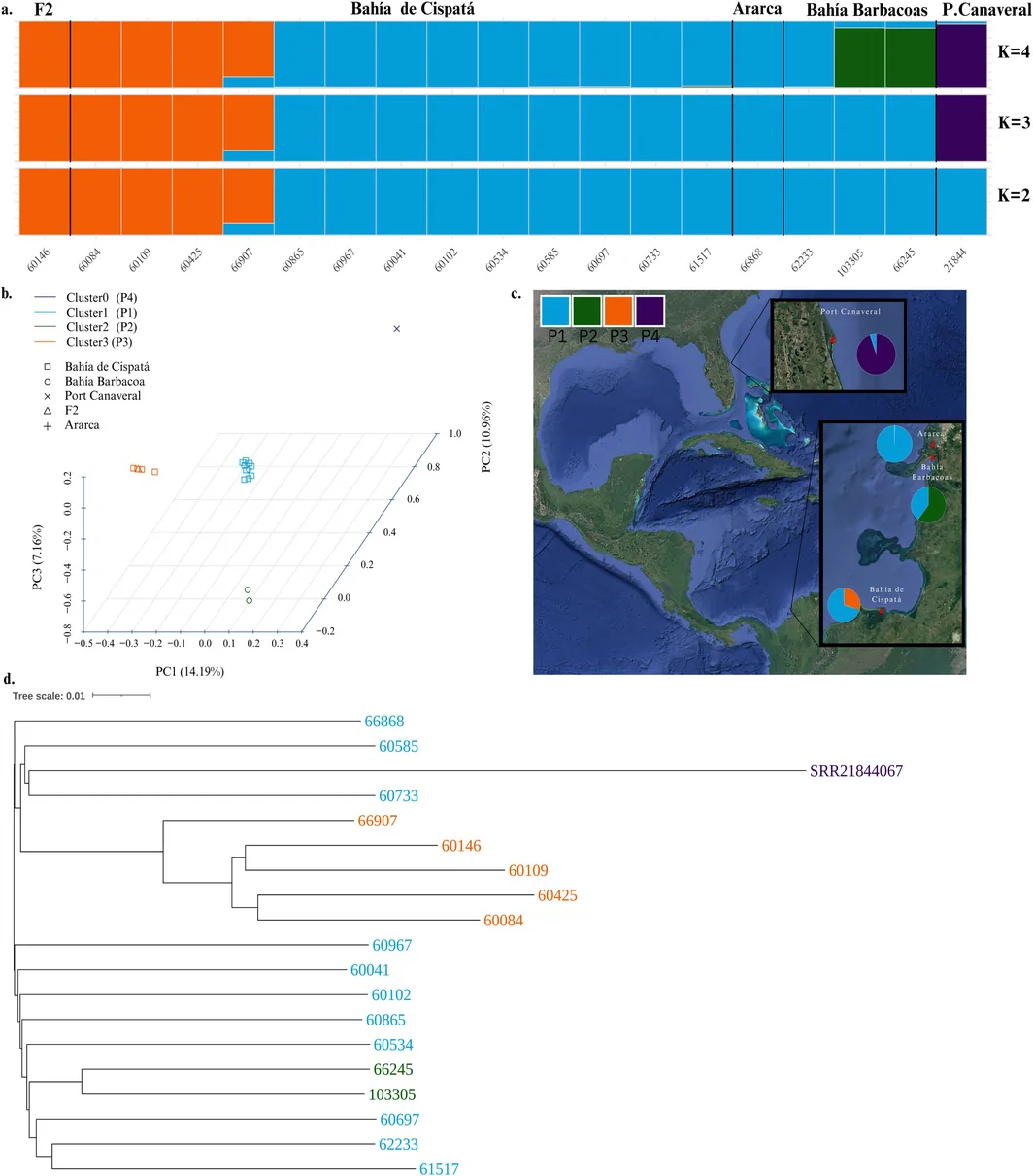
(a) Results of the Structure analysis to determine the genetic assignment of goliath grouper samples in different locations in Colombia and Port Canaveral, Florida, USA. Each bar represents an individual and the colors indicate the proportion of genetic ancestry assigned to each cluster in models with *K* = 2, *K* = 3 y *K* = 4. All localities, except Port Canaveral, correspond to Colombian populations. Sample “F2” comes from the offspring of crosses made in the Oceanario. (b) Principal component analysis (PCA) of the captive population of goliath grouper in the Oceanario against a sample from Puerto Canaveral (x), USA. (c) Genetic clusters distribution of goliath grouper in Colombian localities and east coast of Florida, based on Structure analysis. The pie plots show the proportion of each genetic cluster between different localities: P1 (blue), P2 (green), P3 (orange) and P4 (purple). Basemap imagery from Google Earth (Google, Maxar Technologies). (d) Tree constructed with a *Hierarchical Clustering* analysis using a genetic distance matrix based on 32,813 SNPs. The tree reveals the genetic relationships between the sample of goliath grouper, where the assigned colors of each sample correspond to the dominant ancestry based on Structure results.

Results from Structure, using the Puechmaille test (Puechmaille 2016), indicated that the optimal *K* was four in all metrics, considering the inclusion of the Florida sample. Because the dataset included a limited number of individuals from some sampling locations (Bahía Barbacoas, *n* = 3; Ararca, *n* = 1; Florida, *n* = 1), the inferred clustering patterns should be considered preliminary and interpreted as exploratory. The analysis suggested genetic differentiation among lineages, with only one sample from Bahía de Cispatá showing evidence of admixture (18% shared ancestry from the blue lineage and 82% assigned to the orange lineage), while a low proportion of blue lineage ancestry was observed in Bahía Barbacoas (Figure 4a). The sample from Port Canaveral exhibited some degree of blue lineage ancestry. The captive‐bred F2 individual (ID 60146; Table S1), which does not represent a wild geographic population, was assigned entirely to the orange lineage, indicating that its genetic background is consistent with this lineage. The blue lineage was detected across all sampled locations, although at variable proportions (Figure 4a). Principal component analysis (PCA) further supported these findings, explaining 32.31% of the variance across three principal components (PCs). This analysis revealed substructure within Colombian populations and highlighted their differentiation from the Florida sample (Figure 4b).

Finally, a Neighbor Joining tree revealed relationships based on a distance matrix between individuals. Samples from the orange lineage were distinctly grouped in a separate clade from the other samples. Within the blue lineage, substructure was observed. Additionally, the progeny (ID: 60146) from the crosses carried out at the Oceanario clustered with sample 60,109 (Figure 4d).

Genetic diversity indices estimated from 7706 SNP loci are presented in Table 2 and illustrated in Figures S6 and S7. Cluster Blue (*n* = 11) exhibited higher expected heterozygosity (*H*_e_ = 0.2361 ± 0.0017) and nucleotide diversity (π = 0.2474 ± 0.0018) than Cluster Orange (*n* = 5; *H*_e_ = 0.1593 ± 0.0023; π = 0.1770 ± 0.0025), suggesting greater allelic richness in the former. The orange cluster showed a marked excess of observed over expected heterozygosity (*H*_o_ = 0.2016 ± 0.0032 vs. *H*_e_ = 0.1593 ± 0.0023), consistent with a negative inbreeding coefficient (*F*_IS_ < 0) and a pattern of heterozygote excess. This difference is likely produced by the small sample size taking into account that the minimum *H*_o_ for sites with heterozygous individuals is 0.2 (1/5) in this case. In contrast, the blue cluster showed near‐identity between *H*_o_ (0.2371 ± 0.0020) and *H*_e_ (0.2361 ± 0.0017), indicating conformity with Hardy–Weinberg proportions. These patterns were consistent across loci, as illustrated by the per‐locus distributions in Figure S7. Estimates for the green cluster (*n* = 2) are reported in Table 2 but must be interpreted with caution given the small sample size.

**TABLE 2:** Within‐cluster genetic diversity indices estimated from 7706 SNP loci in the Atlantic Goliath grouper ( *Epinephelus itajara* ) across *K* = 4 genetic clusters identified by STRUCTURE.

Cluster	*n*	*H*_0_ ± SE	*H*_1_ ± SE	π ± SE	Private alleles	φ within
Cluster orange	5	0.2016 ± 0.0032	0.1593 ± 0.0023	0.1770 ± 0.0025	371	0.2048
Cluster blue	11	0.2371 ± 0.0020	0.2361 ± 0.0017	0.2474 ± 0.0018	2096	−0.0016
Cluster green	2	0.2633^a^ ± 0.0039	0.1788^a^ ± 0.0024	0.2384^a^ ± 0.0032	121	0.1387
Cluster purple	1	—	—	—	199	—

*Note:* Values shown as mean ± SE across loci. Dashes (—) indicate that the metric could not be estimated for clusters with *n* = 1. Private Alleles, number of alleles exclusive to each cluster; φ within, mean pairwise kinship coefficient within cluster (VanRaden 2008 moment estimator).

Abbreviations: *H*_0_, observed heterozygosity; *H*_e_, expected heterozygosity; π, nucleotide diversity (unbiased estimator).

^a^ Estimates for Cluster Green (*n* = 2) should be interpreted with caution due to the very small sample size.

Pairwise genetic differentiation between the orange and the blue clusters was moderate‐to‐high (*F*_ST_ = 0.1823 ± 0.0023; Table 3), indicating substantial allele‐frequency divergence between these two groups. *F*_ST_ estimates involving the green cluster were computed but should be treated with caution (*F*_ST_ Orange–Green = 0.0817 ± 0.0046; *F*_ST_ Blue–Green = 0.1375 ± 0.0045) due to the small sample size of that cluster (*n* = 2). *F*_ST_ could not be estimated for comparisons involving the purple cluster (*n* = 1).

**TABLE 3:** Pairwise genetic differentiation (*F*_ST_) and kinship (φ) among *K* = 4 genetic clusters in the Atlantic Goliath grouper ( *Epinephelus itajara* ). Upper‐right triangle: pairwise *F*_ST_ (Nei's GST) ± SE. Diagonal: within‐cluster kinship (φ within). Lower‐left triangle: mean pairwise kinship between clusters (φ).

	Orange (*n* = 5)	Blue (*n* = 11)	Green (*n* = 2)	Purple (*n* = 1)
Orange (*n* = 5)	φ within: 0.2048	*F*_ST_: 0.1823 ± 0.0023	*F*_ST_: 0.0817 ± 0.0046^a^	*F*_ST_: NA^b^
Blue (*n* = 11)	φ: −0.0928	φ within: −0.0016	*F*_ST_: 0.1375 ± 0.0045^a^	*F*_ST_: NA^b^
Green (*n* = 2)	φ: −0.0967	φ: −0.0127	φ within: 0.1387	*F*_ST_: NA^b^
Purple (*n* = 1)	φ: −0.1120	φ: −0.0269	φ: −0.0430	φ within: —

*Note:* *F*_ST_, genetic differentiation index (Nei 1987); φ, kinship coefficient estimated using the genomic relationship matrix (VanRaden 2008). Positive φ values indicate relatedness above the population average; negative values indicate unrelated or outbred pairs relative to the sample mean.

^a^ Estimate involves Cluster Green (*n* = 2) or Cluster Orange (*n* = 5); interpret with caution due to small sample size.

^b^ *F*_ST_ not calculable: at least one cluster has *n* = 1, providing insufficient allele frequency information.

Private allele counts varied considerably among the four *K* = 4 clusters (Table 2 and Figure S8). The blue cluster harbored the largest number of exclusive alleles (2096), followed by the orange cluster (371), the purple cluster (199), and the green cluster (121). It should be noted that the counts for the purple cluster (*n* = 1) and the green cluster (*n* = 2) were conservative underestimates, as only alleles present in the sampled chromosomes could be detected.

Pairwise kinship coefficients (φ) among all 19 individuals are displayed as a color‐coded heatmap in Figure S9 and as within‐ and between‐cluster distributions in Figure S10; summary values are reported in Tables 2 and 3. Within‐cluster kinship was markedly elevated in the orange cluster (φ *within* = 0.2048), a value approaching the expectation for first‐degree relatives (φ ≈ 0.25). This suggests that the five individuals in this cluster shared a large proportion of their genome identical‐by‐descent. Cluster green also showed moderate within‐cluster kinship (φ *within* = 0.1387), consistent with second‐degree relatedness among its two members. In contrast, within‐cluster kinship in the blue cluster was essentially zero (φ *within* = −0.0016), indicating that individuals within this cluster were effectively unrelated relative to the sample‐wide baseline, a pattern typical of natural outbreeding populations. All between‐cluster kinship values were negative (Table 3), reflecting that individuals from different clusters shared fewer alleles than the population‐wide average: the most divergent comparisons were observed between the orange clusters and the other clusters (φ = −0.1120 against purple), (φ = −0.0967 against green) and (φ = −0.0928 against blue). The kinship heatmap (Figure S8) confirmed these patterns visually, with the Cluster Orange block displaying warm red tones (high relatedness) and all cross‐cluster blocks displaying cold blue tones (genetic divergence).

## 4 Discussion

### 4.1 *E. itajara* Genome

In this study, we conducted a reference‐guided chromosome‐scale genome assembly of *E. itajara* . The total genome length of *E. itajara* (1.12 GBp) is nearly the same size as that of *E. malabaricus* (~1.09 Gb; Huerlimann et al. 2024) and *E. lanceolatus* (~1.086 Gb; Zhou et al. 2019), but larger than that of *E. awoara* (~984.48 Mb; Zhang et al. 2024). *E. itajara* has fewer protein‐coding genes (22,692) compared to *E. malabaricus* (26,140; Huerlimann et al. 2024) and *E. awoara* (24,541; Zhang et al. 2024), although *E. lanceolatus* has a similar number of protein‐coding genes (23,974; Zhou et al. 2019).

Collinearity analysis between the genome of *E. itajara* and those of *Epinephelus* species revealed high collinearity across the chromosomes, with the exception of *E. akaara* and *E. moara*. The groupers are commonly used in aquaculture (Muhammadar et al. 2014; Palm et al. 2015), for example brown‐marbled grouper ( *E. fuscoguttatus* ) where females are used as parents for hybridization with other grouper species, such as *E. fuscoguttatus* ♀ × *E. lanceolatus* ♂ (Nankervis et al. 2022), *E. fuscoguttatus* ♀ × potato grouper ( *E. tukula* )♂ (Cheng et al. 2019), and *E. fuscoguttatus* ♀ × *E. polyphekadion* ♂ (Palm et al. 2015; Amenyogbe et al. 2020; Yang et al. 2022). A putative inversion event was observed on chromosome 4 (Figure 2 and Figure S11) of *E. itajara* against all the species. Figure 2a suggests that the two species share most of their gene content, which is promising for identifying genes related to adaptations in captivity. A particular trait of interest is osmoregulation, since the goliath grouper is a euryhaline species capable of surviving in brackish or even freshwater environments (García et al. 2013).

Regarding reproductive behavior, Bullock (1992) determined that the groupers are gonochorist (meaning, male or female) but Smith (cited in South Atlantic Fishery Management Council (SAFMC) 1990) signaled that “most, if not all groupers are protogynous hermaphrodites”. However, the evidence for these theories is contradictory. For example, in the Gulf of Mexico and Bermudas, males have been observed with female gonadal tissue on mature testes (Murie et al. 2023). The protogynous hermaphrodites can be diandric meaning that there are two ways of becoming a male: born as a male or switch from female to male. This type of determination is more resilient to fishing pressures. This is important because if the males are bigger and long‐lived than females, they can become a target for fishing, creating a problem of sperm shortage (Murie et al. 2023). For other species belonging to the subfamily Epinephelinae in general, the ratio of females to males is approximately 4:1 (Claro et al. 1990; Brulé et al. 2003). The sex ratio determined in the study by Freitas (2015) is strongly biased towards females, suggesting that large males are being overexploited in the Abrolhos Bank (Freitas 2015). However, Murie et al. (2023) determined that for *E. itajara* the ratio of males and females was 1:1, suggesting that the sexual determination of this species is still undetermined.

Genome assemblies offer valuable tools for investigating the genes responsible for sexual differentiation. In the context of sexual determination research in the goliath grouper, it is important to consider recent findings on the spotted grouper ( *E. coioides* ). This species serves as an ideal model for studying the molecular mechanisms of sexual differentiation and sex change, as it exhibits a pattern of protogynous hermaphroditism, where most males initially develop as females before undergoing sex reversal. Studies on *E. coioides* have identified both genetic and social signals that influence these processes, which could provide crucial insights for understanding sexual determination in *E. itajara* , a topic that remains unresolved and unclear (Li et al. 2023).

### 4.2 Population Genomics

This study presents the first findings on the genomic diversity of this species in Colombian territory. The analysis of population structure revealed a marked separation among the evaluated samples. A total of four genetic clusters were identified, including a sample from Florida (Figure 4c). Three genetic lineages were suggested within Colombian samples, and it was hypothesized that the orange and green lineages are unique to Bahía de Cispatá and Bahía Barbacoas, respectively. However, these observations should be interpreted with caution because the sampling was highly unbalanced, with only three individuals from Bahía Barbacoas and single representatives from Ararca and Florida. Consequently, the present dataset is insufficient to determine whether the inferred lineages are geographically exclusive, to accurately estimate gene flow among regions, or to define management units. Increasing both the number of individuals and the geographic coverage will be necessary to test these hypotheses.

The within‐cluster diversity metrics also revealed differences in genetic diversity among the inferred groups (Table 2). The blue cluster exhibited higher expected heterozygosity and nucleotide diversity than the orange cluster, suggesting that it retains greater standing genetic variation. In contrast, the excess of observed over expected heterozygosity detected in the orange cluster, together with the elevated within‐cluster kinship, indicates that individuals assigned to this lineage are genetically more similar to one another. However, because the orange cluster was represented by only five individuals, these estimates should be interpreted cautiously until larger population samples become available. Although groupers are highly faithful to their native habitats, migrations of up to 153 km have been observed in Cuba for spawning aggregations (Pina‐Amargós and González‐Sansón 2009). The distance between the town of Ararca and Bahía de Cispatá is approximately 95 km, suggesting that genetic flow between these populations is a possibility (Figure 4c). Unfortunately, there is no official evidence of such aggregations in the Colombian Caribbean waters. However, local fishermen reported that these spawning events had been observed in the past, but they have not been seen in the last 25 years.

Recent genetic studies have demonstrated that goliath grouper populations in the western Atlantic are not demographically connected (Craig et al. 2009). This pattern has been confirmed using mitochondrial and microsatellite markers, which revealed the presence of unique haplotypes distributed unevenly between populations in the United States and Brazil (Vaz‐Perreira et al. 2007). In the Colombian context, the pronounced genetic differentiation between the observed lineages aligns with the pattern of discrete populations in the western Atlantic. Although no clear physical barriers have been identified between the studied localities (e.g., Bahía de Cispatá and Bahía Barbacoas) (Figure 4c), the results suggest some level of genetic differentiation among sampled locations. This pattern may reflect restricted connectivity between sampling sites; however, the current sampling design does not allow robust inference of migration rates or regional connectivity because several localities were represented by very few individuals. Broader geographic sampling with larger sample sizes per locality will be required to determine whether the observed differentiation reflects stable population structure or sampling effects. Possible explanations include philopatric behavior of groupers or the absence of spawning aggregations in recent decades, as reported by local fishermen.

The moderate‐to‐high genetic differentiation observed between the two best‐sampled lineages, together with the presence of numerous private alleles in each cluster (Tables 2 and 3 and Figure S8), is consistent with restricted gene exchange over evolutionary timescales. Nevertheless, because several sampling locations were represented by very few individuals, these patterns should be regarded as preliminary until they can be validated with broader geographic sampling.

These findings provide an initial genomic framework for future conservation and captive breeding efforts of goliath grouper in Colombia. The combination of population structure analyses, within‐cluster diversity estimates, genetic differentiation, and kinship analyses suggests that the sampled individuals do not represent a single homogeneous population. However, broader geographic sampling and larger sample sizes will be required before these lineages can be considered independent management units. Such efforts will be essential for preserving the genetic diversity of Colombian populations and informing future conservation strategies.

### 4.3 Comparative Genomics

Orthogroups of the 9 *Epinephelu*s species were predicted. 205,348 genes were clustered in 20,353 orthogroups (Figure 3b) of which 8685 were single‐copy gene families. Those single‐copy gene families were used to perform a nucleotide evolution analysis by calculating synonymous mutation rates (Ks) between the species (Figure 3a). These results are further supported by mitochondrial genome analyses, which consistently recover *E. lanceolatus* as the sister species of *E. itajara* when compared across 55 mitochondrial genomes from the genus *Epinephelus*, representing 41 species, with multiple sequences available for some taxa (Padgett and Baeza 2025). The low Ks value (~0.01; Figure 3a), supports the recent divergence and close evolutionary relationship between *E. itajara* and *E. lanceolatus* . Based on these estimates, a tentative divergence time of approximately ~4 Mya can be proposed, which is consistent with values reported across multiple genomic studies within the genus. Additionally, fossil evidence from Venezuela attributed to a possible ancestor of *E. itajara* suggests a minimum divergence bound of ~5.3 Mya when compared to *E. lanceolatus* (Ma et al. 2016).

Several studies have reported divergence times within the genus *Epinephelus* using different genomic resources and calibration strategies. For instance, the clade comprising *E. moara*, *E. lanceolatus* , and *E. fuscoguttatus* has been estimated to diverge around 6.28 Mya (Zhao et al. 2025), with *E. lanceolatus* and *E. fuscoguttatus* splitting more recently at approximately 5.57 Mya (Zhao et al. 2025). However, broader divergence ranges have been reported depending on the dataset and methodology. For example, genome assembly analyses of *E. fuscoguttatus* estimated the divergence of the *lanceolatus–fuscoguttatus* clade between 12 and 26 Mya (Yang et al. 2022). Similarly, the divergence between *E. moara* and *E. lanceolatus* has been estimated at ~6.8 Mya (Zhou et al. 2021), and the clade including *E. tukula* and *E. lanceolatus* shows divergence estimates ranging from 9 to 20 Mya (Wang et al. 2022). Consistent with deeper phylogenetic splits within the genus, the genome assembly of *E. cyanopodus* indicates that the clade comprising *E. cyanopodus* and *E. akaara* diverged from the lineage including *E. lanceolatus* , *E. fuscoguttatus* , and *E. moara* approximately 25.8 Ma (Cao et al. 2022), supporting an early to mid‐Miocene diversification for these groups (Ma et al. 2016).

Despite this variability, most recent estimates converge on a mid‐Miocene diversification scenario for the genus *Epinephelus*. This temporal framework suggests that major geological and oceanographic events likely played a key role in shaping current phylogenetic relationships. In particular, the divergence between *E. itajara* and *E. lanceolatus* may be associated with the final stages of the closure of the Isthmus of Panama (~3.2 Mya), which created a major biogeographic barrier between the Atlantic and Pacific Oceans. Consistent with this, mitochondrial data indicate that the Atlantic *E. itajara* and the Pacific lineage (now recognized as *E. quinquefasciatus* ) diverged approximately 3 Mya (Craig et al. 2009), supporting a vicariant speciation scenario driven by this event. Furthermore, divergence estimates between *E. lanceolatus* and *E. itajara* ranging from ~2.9 to 5.5 Mya (Craig et al. 2009) align closely with this geological timeframe.

Interestingly, high genomic collinearity between closely related grouper species has been associated with the potential for hybridization (Nankervis et al. 2022; Cheng et al. 2019; Amenyogbe et al. 2020; Yang et al. 2022). Indeed, hybrids between *E. fuscoguttatus* and *E. lanceolatus* have already been reported, suggesting that reproductive compatibility may be retained among recently diverged lineages. Given the close relationship between *E. itajara* and *E. lanceolatus* , this raises the possibility of hybridization involving *E. itajara* or its Pacific counterpart, *E. quinquefasciatus* , although genomic resources for the latter remain limited. Notably, *E. quinquefasciatus* was historically considered conspecific with *E. itajara* , but genetic evidence has confirmed their separation as distinct species (Craig et al. 2009).

Finally, phylogenomic analyses also place *E. tukula* in close proximity to *E. lanceolatus* and *E. fuscoguttatus* (Wang et al. 2022), which is consistent with the clustering patterns observed in our Ks‐based comparisons (Figure 3a). This supports the hypothesis that *E. tukula* represents one of the closest relatives to the *itajara–lanceolatus* lineage after *E. lanceolatus* itself, further refining the evolutionary framework of this group.

### 4.4 Strategies and Implications for Conservation

Understanding movement patterns and reproductive mechanisms is essential for developing effective conservation strategies. Groupers tend to reproduce at specific times and locations, making them highly predictable and vulnerable to fishing pressure (Murie et al. 2023). Unfortunately, there are no official geographical records of goliath grouper aggregations in Colombia. In Brazil and the United States, however, it is well documented that grouper aggregations increase during full and new moons, with the latter being more favorable for larval dispersal due to reduced luminosity, which helps minimize predation (Bueno et al. 2016; Koenig et al. 2017).

Both Brazil and the United States have implemented fishing moratoriums, leading to an increase in juvenile populations in the U.S. (Bertoncini et al. 2018; Frias‐Torres 2013). However, illegal fishing remains a significant issue in Brazil, with over 300 tons recorded (Freitas 2015). In Colombia, there is no legislation regarding minimum catch sizes, though a zero‐ton quota for groupers is in place for the Colombian Caribbean, except for the Santa Marta marsh (Ciénaga Grande de Santa Marta) (Resolución 334 de 2013, Ministerio de Agricultura y Desarrollo Rural). Establishing minimum catch sizes is a priority to ensure that individuals reach reproductive events before being harvested. A potential minimum size could be at least 140 cm. For instance, in the Gulf of Mexico, males reach sexual maturity between 110 and 115 cm total length (TL), while females mature between 120 and 135 cm TL (Artero et al. 2015). Another study in the Gulf of Mexico found a minimum sexual maturity size of 125 cm (Brule et al. 2018), while in Florida, maturity is reached between 110 and 120 cm (Sadovy and Eklund 1999). In Brazil, 100% of the sampled individuals were sexually mature at 126 cm (Freitas 2015).

Despite substantial evidence of a critical genetic status for this species, the International Union for Conservation of Nature (IUCN) reclassified the goliath grouper as “vulnerable” in 2018 (Bertoncini et al. 2018), down from its previous designation as “critically endangered” (IUCN 1996). Given the ongoing threats to its populations, it is crucial to restore its critically endangered status. The Instituto de Investigaciones Marinas y Costeras José Benito Vives de Andréis (INVEMAR) has already classified the species as critically endangered in Colombia's Red List of Marine Fish (Andrea Polanco et al. 2017). One key conservation measure to ensure genetic connectivity between populations is the establishment of protected fishing zones around suspected aggregation sites and migratory routes. Additionally, monitoring these events could help prevent fishing during key reproductive periods (Bueno et al. 2016; Pina‐Amargós and González‐Sansón 2009). However, identifying these aggregation zones along the Colombian Caribbean coast is a necessary first step. Important actions include investigating the movement patterns of goliath groupers and determining whether their aggregation sites fall within protected areas, such as National Natural Parks. If these sites are not currently protected, prioritizing their designation as conservation zones would be critical for maintaining genetic flow between populations.

Studies conducted in the Gulf of Mexico (Mann et al. 2009) and off the southwest coast of Florida have shown that this species uses low‐frequency acoustic signals during reproductive behavior. Sounds with dominant frequencies around 60 Hz were primarily recorded between 1:00 and 3:00 a.m., with a noticeable lunar periodicity—sound production decreased during full moons. Acoustic telemetry monitoring has revealed that individuals tend to remain at aggregation sites for extended periods, staying near the bottom (46 m) but making vertical excursions to shallower waters around midnight and 3:00 a.m., likely linked to reproductive activity. These findings suggest the potential for a bioacoustics research project in Colombia to identify and monitor reproductive aggregations. The application of passive acoustics and active telemetry could enable the spatial and temporal tracking of critical areas, facilitating the development of effective management and conservation strategies to protect this threatened species.

Finally, the genomic framework generated in this study provides a foundation for the development of genetically informed captive breeding programs at the Oceanario Islas del Rosario. A more comprehensive understanding of the species population structure can be achieved by incorporating additional individuals across the Colombian Caribbean. This will enable the identification of genetically appropriate breeding pairs to maximize the retention of genetic diversity and to minimize the risk of mating closely related individuals. In the longer term, these genomic resources could also support restoration or reinforcement programs, provided that future studies first determine the geographic distribution, connectivity, and conservation relevance of the inferred genetic lineages.

## Author Contributions

**Juan Esteban Jácome Castro:** conceptualization (lead), data curation (lead), formal analysis (lead), funding acquisition (lead), investigation (lead), methodology (lead), project administration (lead), resources (lead), software (lead), validation (lead), visualization (lead), writing – original draft (lead), writing – review and editing (lead). **José Gregorio Martínez:** data curation (equal), formal analysis (equal), methodology (equal), supervision (equal), validation (equal), writing – original draft (equal), writing – review and editing (equal). **Jaime Rojas:** data curation (equal), formal analysis (equal), investigation (equal), methodology (equal), resources (equal), supervision (equal), validation (equal), writing – original draft (equal), writing – review and editing (equal). **Nadine Hakim:** data curation (equal), formal analysis (equal), investigation (equal), methodology (equal), resources (equal), validation (equal), writing – original draft (equal), writing – review and editing (equal). **Diego Alejandro Quiroga:** formal analysis (equal), investigation (equal), methodology (equal), validation (equal), writing – original draft (equal), writing – review and editing (equal). **Jorge Duitama:** data curation (equal), formal analysis (equal), investigation (equal), methodology (equal), resources (equal), software (lead), supervision (lead), validation (equal), visualization (equal), writing – original draft (equal), writing – review and editing (equal). **Susana Caballero:** data curation (equal), formal analysis (equal), investigation (equal), methodology (equal), resources (lead), supervision (lead), validation (equal), visualization (equal), writing – original draft (equal), writing – review and editing (equal).

## Funding

This project was funded entirely with funds from the PADI Foundation under grant #89748 (to J. E. Jácome). The authors would like to thank the Vice Presidency of Research & Creation’s Publication Fund at Universidad de los Andes for its financial support.

## Conflicts of Interest

The authors declare no conflicts of interest.

## Supporting information


**Data S1:** Supporting information.

**Table S1:** Additional information about samples. TL, total length; FL, fork length. The F2 individual corresponds to the surviving offspring from the second captive breeding attempt carried out at the Oceanario and was included only to assess its genomic affinity relative to the inferred lineages; it should not be interpreted as representing a natural population sample. Individual 103,305 was recaptured during the second sampling campaign and therefore appears twice in this table because morphometric measurements were recorded on both capture occasions. However, it was sequenced only once, and only one sequencing dataset from this individual was included in all population genomic analyses.
**Table S2:** List of grouper species (genus *Epinephelus*) with fully assembled genomes available in public databases. The table includes the common name, scientific name, database source, and genome accession number.
**Figure S1:** Estimation of the genome size of *Epinephelus itajara* using *k*‐mers analysis with GenomeScope. A *k*‐mer size of 21 was used, from Illumina WGS sequencing data. The estimated length of the genome is 1.01 Gb, with a unique sequence content of 77.8%. The average coverage (kcov) was 35.3×, with an error rate of 0.208% and a duplication level of 1.81. Dashed lines indicate the coverage peaks of the *k*‐mers used to model the distribution. The observed curve is depicted in blue, while the fitted model is outlined in black.
**Figure S2:** Circular representation of the mitochondrial genome of *Epinephelus itajara* . Colors define the orientation of the annotated segments (blue for positive, red for negative).
**Figure S3:** BUSCO results for the Actinopterygii class. The bar shows the completeness of the genome assembled.
**Figure S4:** Merqury *k*‐mer copy number spectra of the final assembly showing the distribution of *k*‐mers across assembly copy numbers.
**Figure S5:** Coverage‐depth distribution obtained by mapping PacBio HiFi reads back to the final genome assembly using Minimap2 and calculating per‐base read depth with Samtools.
**Figure S6:** Comparison of within‐cluster genetic diversity indices estimated from 7706 SNP loci. Mean observed heterozygosity (*H*_o_), expected heterozygosity (*H*_e_), and nucleotide diversity (π) are shown for the inferred genetic clusters with sample sizes ≥ 5 individuals (Cluster Orange, *n* = 5; Cluster Blue, *n* = 11). Error bars indicate the standard error across loci.
**Figure S7:** Distribution of per‐locus genetic diversity indices for the two inferred genetic clusters with sample sizes ≥ 5 individuals. Violin plots depict the distributions of observed heterozygosity (*H*_o_), expected heterozygosity (*H*_e_), and nucleotide diversity (π) across 7706 SNP loci. Dots correspond to individual loci, and the embedded boxplots indicate the median and interquartile range.
**Figure S8:** Private alleles identified among the inferred genetic clusters. Bars represent the number of alleles occurring exclusively within each genetic cluster across 7706 SNP loci.
**Figure S9:** Pairwise genomic kinship coefficients among the 19 sampled individuals. Individuals are grouped according to their inferred genetic clusters. Colors represent the genomic kinship coefficient (φ), with warmer colors indicating greater relatedness and cooler colors indicating lower or negative relatedness. Black lines delimit inferred genetic clusters.
**Figure S10:** Distribution of pairwise genomic kinship coefficients (φ) within and between inferred genetic clusters. Each point represents a pairwise comparison between individuals. Positive φ values indicate greater genomic relatedness, whereas values close to zero or below zero indicate unrelated or genetically divergent individuals relative to the study population.
**Figure S11:** Whole‐chromosome dot plots used to evaluate the putative inversion identified on chromosome 4 of *E. itajara* . Each panel contains two pairwise comparisons using the original contigs contributing to chromosome 4 of *E. itajara* as the reference. (a) Upper: *E. lanceolatus* chromosome 4; lower: *E. fuscoguttatus* chromosome 5. (b) Upper: *E. awoara* chromosome 4; lower: *E. polyphekadion* chromosome 6. (c) Upper: *E. tukula* chromosome 7; lower: *E. cyanopodus* chromosome 5. (d) Upper: *E. akaara* chromosome 7; lower: *E. moara* chromosome 2. These comparisons were performed to assess whether the rearrangement detected in the synteny analysis is consistent with a putative chromosomal inversion.
**Figure S12:** Whole‐chromosome dot plots used to evaluate the putative inversion identified on chromosome 5 of *E. itajara* . Each panel contains two pairwise comparisons using the original contigs contributing to chromosome 5 of *E. itajara* as the reference. (a) Upper: *E. lanceolatus* chromosome 5; lower: *E. fuscoguttatus* chromosome 4. (b) Upper: *E. awoara* chromosome 5; lower: *E. polyphekadion* chromosome 4. (c) Upper: *E. tukula* chromosome 4; lower: *E. cyanopodus* chromosome 4. (d) Upper: *E. akaara* chromosome 5; lower: *E. moara* chromosome 20. These comparisons were performed to assess whether the rearrangement detected in the synteny analysis is consistent with a putative chromosomal inversion.
**Figure S13:** Whole‐chromosome dot plots used to evaluate the putative inversion identified on chromosome 19 of *E. itajara* . Each panel contains two pairwise comparisons using the original contigs contributing to chromosome 19 of *E. itajara* as the reference. (a) Upper: *E. lanceolatus* chromosome 19; lower: *E. fuscoguttatus* chromosome 18. (b) Upper: *E. awoara* chromosome 19; lower: *E. polyphekadion* chromosome 19. (c) Upper: *E. tukula* chromosome 19; lower: *E. cyanopodus* chromosome 19. (d) Upper: *E. akaara* chromosome 19; lower: *E. moara* chromosome 9. These comparisons were performed to assess whether the rearrangement detected in the synteny analysis is consistent with a putative chromosomal inversion.

## Data Availability

The genome assembly has been deposited in NCBI under the accession number GCA_055440715.1. Raw sequencing reads generated for this study have been deposited in the National Center for Biotechnology Information (NCBI) under BioProject PRJNA1338326. Individual samples are registered under BioSample accessions SAMN52585810–SAMN52585826 and SAMN52403715, and the corresponding raw reads are available in the Sequence Read Archive (SRA) under accessions SRR36198771–SRR36198789. Additionally, publicly available *E. itajara* reads from Port Canaveral, Florida (SRA/GenBank accession: SRR21844067) were included in the population structure analyses. Supplementary genomic resources associated with this study have been deposited in the Dryad Digital Repository under DOI: 10.5061/dryad.xgxd254wt. The repository includes CDS, cDNA, and protein FASTA files, the final GFF3 genome annotation, custom transposable element libraries, genomic density tracks, and annotation summary statistics derived from the reference‐guided chromosome‐scale genome assembly of *E. itajara* . *Code Availability*: All bioinformatic analyses were performed using the default workflows and command‐line options provided by the software packages described in the Methods section, except where specific parameters are explicitly indicated. The custom Python script used to estimate genetic diversity indices (observed heterozygosity, expected heterozygosity, nucleotide diversity, pairwise *F*_ST_, private allele counts, and genomic kinship) from the STRUCTURE‐format genotype matrix is provided as Supporting Information.
