# Supplementary material for: Reference‐Guided Chromosome‐Scale Genome Assembly With Insights on Population Genomics of the Atlantic Goliath Grouper (Epinephelus itajara), Islas del Rosario, Colombia
Source: Ecol Evol. 2026 Sep 10;16(9):e74323. doi: 10.1002/ece3.74323 (PMC13561635; doi:10.1002/ece3.74323)
Supplement: Supplementary file 1 [file ECE3-16-e74323-s002.docx]

Supplementary tables and figures for:

**Reference-guided chromosome-scale genome assembly with insights on population genomics of the Goliath grouper (*Epinephelus itajara*) Islas del Rosario, Colombia**

Juan Esteban Jácome Castro^1*^, Jose Gregorio Martínez^3^, Jaime Alberto Rojas^2^, Nadine Hakim^1^, Diego Alejandro Quiroga^1^, Jorge Duitama^4*,^ Susana Caballero Gaitán ^1,5*^

^1^Laboratorio de Ecología Molecular de Vertebrados Acuáticos, LEMVA, Departamento de Ciencias Biológicas, Universidad de los Andes, Carrera 1 No. 18ª-10, Bogotá, Colombia

^2^Centro de Investigaciones, Educación y Recreación de las Islas del Rosario (CEINER)

^3^Grupo de Investigación Biociencias, Facultad de Ciencias de la Salud, Institución Universitaria Colegio Mayor de Antioquia.

^4^Systems and Computing Engineering Department, Universidad de los Andes, Bogotá, Colombia. ja.duitama@uniandes.edu.co.

^5^Aquatic Vertebrate Integrative Conservation Laboratory, Marine and Environmental Sciences Department, Guy Harvey Oceanographic Center, Halmos College of Arts and Sciences, Nova Southeastern University, Dania Beach, FL, 33004, USA.

*Corresponding authors; Juan Esteban Jácome Castro. E-mail: [juanestebanjacome4@gmail.com](mailto:juanestebanjacome4@gmail.com) | Jorge Duitama. E-mail: [ja.duitama@uniandes.edu.co](mailto:ja.duitama@uniandes.edu.co) | Susana Caballero Gaitán. E-mail: [scaball1@nova.edu](mailto:scaball1@nova.edu)

Susana Caballero and Jorge Duitama should be considered joint senior authors.

**Table S1.** Additional information about samples. TL: Total Length; FL: Fork Length. The F2 individual corresponds to the surviving offspring from the second captive breeding attempt carried out at the Oceanario and was included only to assess its genomic affinity relative to the inferred lineages; it should not be interpreted as representing a natural population sample. Individual 103305 was recaptured during the second sampling campaign and therefore appears twice in this table because morphometric measurements were recorded on both capture occasions. However, it was sequenced only once, and only one sequencing dataset from this individual was included in all population genomic analyses.

| **ID** | **Weight (kg)** | **TL (cm)** | **FL (cm)** | **Origin** |
| --- | --- | --- | --- | --- |
| **66245** | 12,7 | 85 | 70 | Bahía Barbacoas, Santa Ana, Bolívar, Colombia |
| **103305** | 23,55 | 105 | 89 | Bahía Barbacoas, Santa Ana, Bolívar, Colombia |
| **103305** | 28,65 | 113 | 95 | Bahía Barbacoas, Santa Ana, Bolívar, Colombia |
| **62233** | 47,65 | 134 | 111 | Bahía Barbacoas, Santa Ana, Bolívar, Colombia |
| **66868** | 5 | 62,5 | 51 | Ararca, Bolívar, Colombia |
| **60109** | 18,45 | 95 | 80 | Bahía de Cispatá, Córdoba, Colombia |
| **60967** | 25,95 | 107 | 93 | Bahía de Cispatá, Córdoba, Colombia |
| **60585** | 17,25 | 97 | 82 | Bahía de Cispatá, Córdoba, Colombia |
| **60102** | 22,4 | 107 | 96 | Bahía de Cispatá, Córdoba, Colombia |
| **60733** | 14,25 | 87 | 76 | Bahía de Cispatá, Córdoba, Colombia |
| **60534** | 21,5 | 97 | 83 | Bahía de Cispatá, Córdoba, Colombia |
| **60041** | 26,9 | 104 | 87 | Bahía de Cispatá, Córdoba, Colombia |
| **60697** | 19,7 | 105 | 90 | Bahía de Cispatá, Córdoba, Colombia |
| **60425** | 29,25 | 110 | 95 | Bahía de Cispatá, Córdoba, Colombia |
| **60865** | 32,6 | 117 | 99,5 | Bahía de Cispatá, Córdoba, Colombia |
| **60084** | 28,45 | 105 | 89 | Bahía de Cispatá, Córdoba, Colombia |
| **66907** | 66,15 | 143 | 122 | Bahía de Cispatá, Córdoba, Colombia |
| **61517** | 55,5 | 145 | 128 | Bahía de Cispatá, Córdoba, Colombia |
| **60146** | 29,65 | 110 | 92 | F2 |
| **SRR21844067** |  |  |  | Puerto Cañaveral, Florida, USA |

**Table S2.** List of grouper species (genus *Epinephelus*) with fully assembled genomes available in public databases. The table includes the common name, scientific name, database source, and genome accession number.

| **Common Name** | **Scientific Name** | **Database** | **Accession Number** |
| --- | --- | --- | --- |
| Goliath grouper | *E. itajara* | GenBank | GCA_055440715.1 |
| Giant grouper | *E. lanceolatus* | RefSeq | GCF_041903045.1 |
| Brown-marbled grouper | *E. fuscoguttatus* | RefSeq | GCF_011397635.1 |
| Yellow grouper | *E. awoara* | GenBank | GCA_035609425.1 |
| Longtooth grouper | *E. bruneus* | GenBank | GCA_041684075.1 |
| Kelp grouper | *E. moara* | RefSeq | GCF_006386435.1 |
| Potato grouper | *E. tukula* | GenBank | GCA_021130775.1 |
| Camouflage grouper | *E. polyphekadion* | GenBank | GCA_041411175.1 |
| Speckled blue grouper | *E. cyanopodus* | GenBank | GCA_026686955.1 |
| Orange-spotted grouper | *E. coioides* | GenBank | GCA_900536245.1 |
| Malabar grouper | *E.malabaricus* | GenBank | GCA_037127435.1 |
| Rankin Cod | *E. rankini* | GenBank | GCA_048129235.1 |
| Red spotted grouper | *E. akaara* | Dryad | <https://doi.org/10.5061/dryad.4398b9f> |

**Figure S1.** Estimation of the genome size of Epinephelus itajara using k-mers analysis with GenomeScope. A k-mer size of 21 was used, from Illumina WGS sequencing data. The estimated length of the genome is 1.01 Gb, with a unique sequence content of 77.8%. The average coverage (kcov) was 35.3X, with an error rate of 0.208% and a duplication level of 1.81. Dashed lines indicate the coverage peaks of the k-mers used to model the distribution. The observed curve is depicted in blue, while the fitted model is outlined in black.

**Figure S2.** Circular representation of the mitochondrial genome of Epinephelus itajara. Colors define the orientation of the annotated segments (blue for positive, red for negative).

**Figure S3.** BUSCO results for the Actinopterygii class. The bar shows the completeness of the genome assembled.

**Figure S4.** Merqury k-mer copy number spectra of the final assembly showing the distribution of k-mers across assembly copy numbers.

**Figure S5.** Coverage-depth distribution obtained by mapping PacBio HiFi reads back to the final genome assembly using Minimap2 and calculating per-base read depth with Samtools.

**Figure S6.** Comparison of within-cluster genetic diversity indices estimated from 7,706 SNP loci. Mean observed heterozygosity (H_o_), expected heterozygosity (H_e_), and nucleotide diversity (π) are shown for the inferred genetic clusters with sample sizes ≥ 5 individuals (Cluster Orange, n = 5; Cluster Blue, n = 11). Error bars indicate the standard error across loci.

**Figure S7.** Distribution of per-locus genetic diversity indices for the two inferred genetic clusters with sample sizes ≥ 5 individuals. Violin plots depict the distributions of observed heterozygosity (H_o_), expected heterozygosity (H_e_), and nucleotide diversity (π) across 7,706 SNP loci. Dots correspond to individual loci, and the embedded boxplots indicate the median and interquartile range.

**Figure S8.** Private alleles identified among the inferred genetic clusters. Bars represent the number of alleles occurring exclusively within each genetic cluster across 7,706 SNP loci.

**Figure S9.** Pairwise genomic kinship coefficients among the 19 sampled individuals. Individuals are grouped according to their inferred genetic clusters. Colors represent the genomic kinship coefficient (φ), with warmer colors indicating greater relatedness and cooler colors indicating lower or negative relatedness. Black lines delimit inferred genetic clusters.

**Figure S10.** Distribution of pairwise genomic kinship coefficients (φ) within and between inferred genetic clusters. Each point represents a pairwise comparison between individuals. Positive φ values indicate greater genomic relatedness, whereas values close to zero or below zero indicate unrelated or genetically divergent individuals relative to the study population.

**Figure S11.** Whole-chromosome dot plots used to evaluate the putative inversion identified on chromosome 4 of *E. itajara*. Each panel contains two pairwise comparisons using the original contigs contributing to chromosome 4 of *E. itajara* as the reference. (a) Upper: *E. lanceolatus* chromosome 4; lower: *E. fuscoguttatus* chromosome 5. (b) Upper: *E. awoara* chromosome 4; lower: *E. polyphekadion* chromosome 6. (c) Upper: *E. tukula* chromosome 7; lower: *E. cyanopodus* chromosome 5. (d) Upper: *E. akaara* chromosome 7; lower: *E. moara* chromosome 2. These comparisons were performed to assess whether the rearrangement detected in the synteny analysis is consistent with a putative chromosomal inversion.

**Figure S12.** Whole-chromosome dot plots used to evaluate the putative inversion identified on chromosome 5 of *E. itajara*. Each panel contains two pairwise comparisons using the original contigs contributing to chromosome 5 of *E. itajara* as the reference. (a) Upper: *E. lanceolatus* chromosome 5; lower: *E. fuscoguttatus* chromosome 4. (b) Upper: *E. awoara* chromosome 5; lower: *E. polyphekadion* chromosome 4 . (c) Upper: *E. tukula* chromosome 4; lower: *E. cyanopodus* chromosome 4. (d) Upper: *E. akaara* chromosome 5; lower: *E. moara* chromosome 20. These comparisons were performed to assess whether the rearrangement detected in the synteny analysis is consistent with a putative chromosomal inversion.

**Figure S13.** Whole-chromosome dot plots used to evaluate the putative inversion identified on chromosome 19 of *E. itajara*. Each panel contains two pairwise comparisons using the original contigs contributing to chromosome 19 of *E. itajara* as the reference. (a) Upper: *E. lanceolatus* chromosome 19; lower: *E. fuscoguttatus* chromosome 18. (b) Upper: *E. awoara* chromosome 19; lower: *E. polyphekadion* chromosome 19. (c) Upper: *E. tukula* chromosome 19; lower: *E. cyanopodus* chromosome 19. (d) Upper: *E. akaara* chromosome 19; lower: *E. moara* chromosome 9. These comparisons were performed to assess whether the rearrangement detected in the synteny analysis is consistent with a putative chromosomal inversion.
